# Nurse-Led Nutritional Screening and Early Identification of Malnutrition Among Hospitalized Older Adults: A Scoping Review

**DOI:** 10.3390/nu18183079

**Published:** 2026-09-20

**Authors:** Ayman Mohamed El-Ashry, Abdelaziz Hendy, Saleh M. Alhirsan, Jozaa Z. AlTamimi, Reham I. Alagal, Lujain A. AlMousa, Nora A. AlFaris

**Affiliations:** 1 Psychiatric Mental Health Nursing, Faculty of Nursing, Alexandria University, Alexandria 21615, Egypt; ayman.el-ashry@alexu.edu.eg; 2 Department of Nursing, College of Applied Medical Sciences, Jouf University, Al-Qurayyat 77441, Saudi Arabia; 3 Department of Maternal and Child Health Nursing, College of Nursing, Qassim University, Buraydah 52571, Saudi Arabia; a.hendy@qu.edu.sa; 4 Department of Physical Therapy and Health Rehabilitation, College of Applied Medical Sciences, Jouf University, Al-Qurayyat 77441, Saudi Arabia; smalhersan@ju.edu.sa; 5 Department of Sports Health, College of Sports Sciences & Physical Activity, Princess Nourah bint Abdulrahman University, P.O. Box 84428, Riyadh 11671, Saudi Arabia; jzaltamimi@pnu.edu.sa (J.Z.A.); naalfaris@pnu.edu.sa (N.A.A.); 6 Department of Health Sciences, College of Health and Rehabilitation Sciences, Princess Nourah bint Abdulrahman University, P.O. Box 84428, Riyadh 11671, Saudi Arabia; rialagal@pnu.edu.sa

**Keywords:** older adults, geriatric nursing, hospitalization, malnutrition, nutritional screening, MNA, MST, MUST

## Abstract

**Background/Objectives:** Malnutrition is common in hospitalized older adults and is linked to functional decline, longer stay, readmission, and mortality. Nurses usually screen at admission, but evidence on nurse-led screening is scattered. This review mapped instruments, measurement properties, recognition and referral outcomes, and implementation barriers. **Methods:** A rapid scoping review followed the Joanna Briggs Institute methodology, PRISMA-ScR reporting, and the Population–Concept–Context framework. PubMed, Scopus, and Web of Science Core Collection were searched in June 2026 for studies published from 1 January 1999 to 31 July 2026; in July 2026, the evidence set underwent bibliographic and indexing verification only, with no search update. Two coauthors searched, screened, and charted the data; two others verified all decisions against full texts. Implementation findings were coded inductively without critical appraisal. **Results:** Of 1743 records, 1169 were screened and 18 included. Tools: MNA, MNA-SF, MST, MUST, NRS-2002, SNAQ. Standardized screening improved recognition; undernutrition identification rose from 38.6% to 73.1%. Nurse-administered MST reached 94% sensitivity and 89% specificity in a high-readmission-risk cohort, whereas nurse MNA-SF scoring showed ICC = 0.74 with only fair agreement (kappa = 0.37). Barriers included inconsistent scoring, limited nutrition knowledge, workload, and documentation gaps. **Conclusions:** Nurse-led screening is feasible and improves case finding, recognition, screening completion, and dietitian referral, although scoring errors and inter-rater disagreement persisted. No study measured complications, length of stay, readmission, functional decline, or mortality, so the evidence supports better identification, not better outcomes. Reported accuracy came from selected populations and single assessors. Future work should link screening fidelity to patient outcomes.

## 1. Introduction

Malnutrition and the risk of malnutrition remain common among older people receiving hospital care. A meta-analysis using the Mini Nutritional Assessment (MNA) estimated a malnutrition prevalence of approximately 22% in hospital settings, with a further substantial proportion classified as at nutritional risk [1]. In older adults, acute illness rarely arrives on its own. It is layered on multimorbidity, frailty, polypharmacy, impaired appetite, dysphagia, cognitive impairment, functional dependence, and social vulnerability. Together, these factors reduce dietary intake, accelerate catabolism, and make nutritional deterioration during an admission clinically consequential. Current European Society for Clinical Nutrition and Metabolism (ESPEN) guidance therefore treats the systematic identification of nutritional risk as a core component of geriatric care rather than an optional addition [2].

Nutritional screening is not the same as a diagnosis of malnutrition. Screening is intended to flag patients who are malnourished or at risk and who need further assessment or intervention. Diagnostic frameworks such as the Global Leadership Initiative on Malnutrition (GLIM) set out a two-step pathway in which validated screening is followed by assessment and diagnostic grading [3]. In hospital practice, screening must be rapid, repeatable, feasible at the bedside, and tied to a clear response. The instruments used most often are the MNA and its Short Form (MNA-SF), the Malnutrition Screening Tool (MST), the Malnutrition Universal Screening Tool (MUST), and Nutritional Risk Screening 2002 (NRS-2002) [4,5,6,7,8].

Nurses occupy a strategically important position in this pathway. Admission assessment, routine observation, documentation, and repeated bedside contact all fall within nursing workflows, which makes the nurse the clinician most likely to notice a nutritional problem first. Placing a screening instrument in a nursing admission form, however, does not guarantee accurate or actionable case finding. Early work showed that nurses and dietitians could classify the same older patients differently while using the same locally developed tool [9,10]. More recent studies continue to report scoring discrepancies, weak categorical agreement, incomplete documentation, and high-risk scores that never trigger a referral or a change in care [11,12].

The literature on this topic is heterogeneous in both purpose and design. Some studies validate nurse-administered screening against a reference assessment. Others compare standardized tools with nursing judgement, audit screening completion, or examine nurses’ knowledge, attitudes, role perceptions, documentation practices, and organizational constraints. A conventional systematic review restricted to diagnostic accuracy would capture only part of this picture and would leave the implementation evidence out. Scoping reviews are designed for exactly this situation, since they identify and describe the breadth, characteristics, concepts, and gaps within a heterogeneous evidence base [13,14,15]. A scoping approach was therefore selected so that direct screening-performance evidence and implementation evidence could be mapped alongside each other.

## 2. Aim, Objectives, and Review Questions

The aim of this scoping review was to map and synthesize the available evidence on nurse-led nutritional screening and the early identification of malnutrition among hospitalized older adults.

The objectives were to: (1) identify the screening tools and recognition approaches used by nurses; (2) describe measurement properties and case-finding outcomes where these were reported; (3) map the barriers and facilitators affecting screening, documentation, referral, and follow-up; and (4) identify the methodological and practice gaps that should guide future research and quality improvement.

The review addressed four questions:

1. What nutritional screening or early-identification approaches have been used by practicing nurses for hospitalized older adults?
2. What evidence is available regarding reliability, validity, diagnostic accuracy, recognition, screening completion, or referral following nurse-led screening?
3. What individual, professional, workflow, and organizational factors facilitate or hinder nurse-led nutritional screening and early identification?
4. What gaps remain in the evidence regarding nurse-specific screening performance and downstream clinical outcomes?

## 3. Methods

### 3.1. Design and Methodological Framework

A scoping review design was chosen because the review questions call for a broad description of a heterogeneous evidence base rather than a pooled estimate of intervention effectiveness. The review followed the Joanna Briggs Institute (JBI) guidance for scoping reviews where applicable [15,16] and is reported in accordance with the PRISMA Extension for Scoping Reviews (PRISMA-ScR) [17]. Study selection is presented using the PRISMA 2020 flow diagram [18].

**Figure 1:**
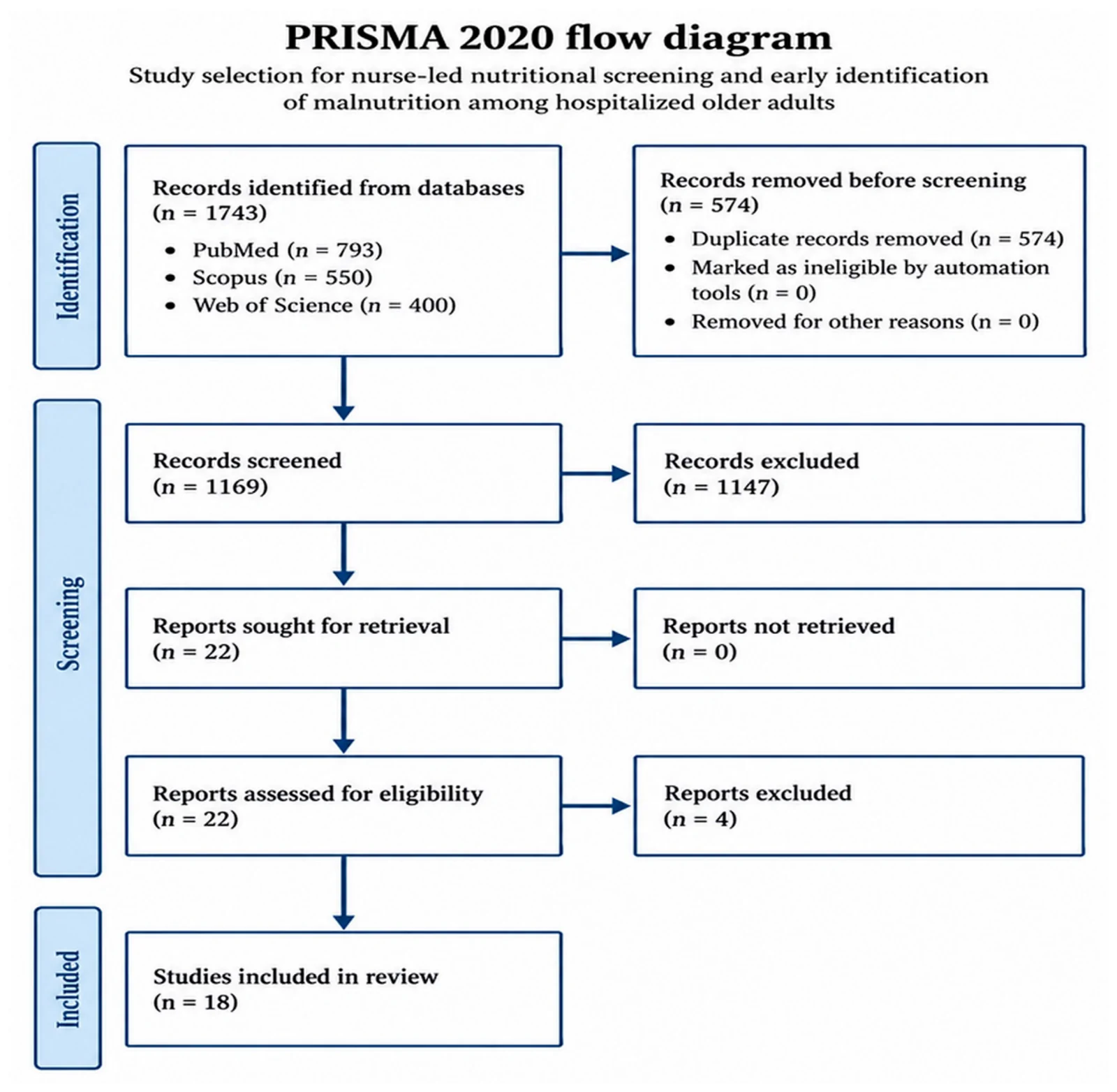
PRISMA 2020 flow diagram of the study-selection process.

A rapid approach was taken to searching, screening, and charting, while the eligibility criteria and the selection process were kept explicit. Three bibliographic databases were searched: MEDLINE via PubMed, Scopus, and Web of Science Core Collection. Each database was searched separately, and the retrieved records were then merged and deduplicated. All database searches were executed in June 2026, and search coverage was limited to records available on the respective search dates. The final evidence set was verified in July 2026; this verification did not constitute a database search update or extend search coverage. Across the three databases, 1743 records were retrieved (793 from PubMed, 550 from Scopus, and 400 from Web of Science). After 574 duplicates were removed, 1169 records proceeded to title and abstract screening; 1147 were excluded, and 22 reports were assessed in full text. Four reports were excluded at that stage, leaving a final evidence set of 18 studies. Database-specific retrieval counts, the deduplication total, the search dates, and the contribution of the verification step are reported in full in Section 3.3, and the selection process is shown in the PRISMA 2020 flow diagram (Figure 1).

Full-text review was carried out for every potentially relevant report that passed title and abstract screening. Selection was based on the predefined Population–Concept–Context (PCC) criteria. For each report, the reviewers confirmed that the population was hospitalized older adults, that practicing nurses had a substantial role in nutritional screening or early identification, and that the report was primary empirical research.

### 3.2. Eligibility Criteria: Population–Concept–Context Framework

Eligibility criteria were structured according to the Population–Concept–Context (PCC) framework. Studies had to satisfy all three PCC elements together with the specified evidence-type, language, and date criteria. The operational inclusion and exclusion criteria are presented in Table 1. The date limits shown in Table 1 are the same limits applied in the database searches described in Section 3.3 and reproduced in Appendix A.

### 3.3. Information Sources and Search Approach

Three bibliographic databases were searched: MEDLINE via PubMed, Scopus, and Web of Science Core Collection. Each was searched separately so that the widest feasible set of relevant studies on nurse-led nutritional screening in hospitalized older adults would be retrieved.

The search strategy was built around five conceptual blocks drawn from the PCC structure and the review question: the older adult population, the inpatient hospital context, malnutrition or nutritional risk, the nursing role, and the screening or early-identification process. Key terms included older adult*, older patient*, elderly, geriatric*, and aged for the population; hospital*, inpatient*, medical ward*, surgical ward*, and acute care for the context; malnutrition, undernutrition, nutritional risk, and nutrition risk for the condition; nurse*, nursing, nurse-led, and nurse-administered for the profession; and screen*, identif*, recogn*, assess*, case finding, admission assessment, document*, referral, nutrition care, barrier*, facilitator*, and implement* for the process. Tool names (MNA, MNA-SF, MST, MUST, NRS-2002, SNAQ) were also included. Database-specific controlled vocabulary, field codes, and syntax were applied where available.

For PubMed, Medical Subject Headings (MeSH) and free-text terms were combined across all five concept blocks. For Scopus and Web of Science, the strategies were applied to the title, abstract, and keyword fields using TITLE-ABS-KEY and Topic, respectively. Synonyms were combined with OR within each block, and the five blocks were combined with AND. The complete strategy for each database is reported in Appendix A so that the search can be reproduced. The publication-date eligibility window was 1 January 1999 to 31 July 2026, and no study-design filter was applied at the database stage. A deliberately sensitive search was preferred, with ineligible evidence types removed at the screening stage. All database searches were executed in June 2026. Accordingly, search coverage was limited to records available on the respective search dates, irrespective of the upper publication-date limit. In July 2026, the final evidence set underwent bibliographic and indexing verification only. The database searches were not rerun or updated, and this verification did not extend search coverage.

The searches returned 1743 records before deduplication: 793 from PubMed, 550 from Scopus, and 400 from Web of Science. Merging the three sets and removing 574 duplicates left 1169 unique records for title and abstract screening. Of these, 1147 were excluded, and 22 reports were retrieved for full-text assessment. Four reports were excluded at the full-text stage because the nursing role in screening was not explicit, because older-adult data could not be extracted separately, or because the report was not primary empirical research. Eighteen studies formed the final evidence set.

The publication details of every included study, including authors, year, journal, volume, pages, and DOI, were checked against the bibliographic databases or the publisher record, and each study was confirmed as indexed in at least one of the three databases searched. No formal backward or forward citation searching was undertaken. This verification step confirmed indexing and bibliographic accuracy rather than seeking new records, and it therefore contributed no additional studies to the evidence set. All 18 included studies were identified through the database searches described above.

### 3.4. Evidence Selection

Candidate records were assessed against the PCC criteria. Titles and abstracts were used at the first pass, and full texts were retrieved for every record that remained plausibly eligible. Studies in which screening was performed by nursing students alone were excluded, because the review concerns practicing nurses and nursing staff. Mixed-setting or mixed-age studies were retained only where the eligible inpatient or older-adult component could be extracted separately. Studies in which the nursing role was not explicit were excluded rather than assumed to be nurse-led.

Two coauthors performed the database searches and the initial title, abstract, and full-text screening. Two further coauthors then checked every retained and excluded full-text report against the PCC criteria and revised the inclusion decisions where required. Disagreements were resolved by discussion among these four coauthors until consensus was reached. This was a sequential review-and-revision workflow rather than fully independent duplicate screening, so no inter-reviewer agreement statistic was calculated. This choice is stated as a limitation in Section 5.6.

### 3.5. Data Charting and Variables

A structured data-charting form was used to capture study-level information. The charted fields were citation details, database source, country and hospital setting, study design, sample characteristics and the age definition used, the nutritional screening or early-identification method, the specific nurse-led component, the reference standard or comparator where applicable, key quantitative or qualitative findings, and any reported implementation barriers or facilitators. Full texts were obtained and read for all 18 included studies.

The two coauthors who conducted the database searches and the initial screening completed the first version of the charting form. The other two coauthors then read the full texts and checked every charted entry against the source article, revising entries where necessary. Queries about sample size, age eligibility, screening method, diagnostic-accuracy statistics, study setting, and the extent of the nursing role were resolved by returning to the published report and discussing the entry until consensus was reached. Data charting therefore involved four coauthors, working in a review-and-revision sequence rather than in independent duplicate extraction.

The final chart covers the 18 studies retained after full-text assessment. Indexing in PubMed, Scopus, and Web of Science Core Collection was verified for each study, and DOI, PMID, Scopus EID, journal, volume, and page details were checked against the corresponding database or publisher record.

### 3.6. Measurement Domains

The 18 included studies measured a wide range of outcomes using diverse methods. They comprise validation and diagnostic-accuracy studies, an inter-rater reliability study, observational and cross-sectional studies, quality-improvement and implementation projects, nurse surveys, and qualitative studies. The measurement domains charted were therefore the prevalence or classification of nutritional risk; sensitivity, specificity, and predictive values against a stated reference standard; inter-rater reliability, expressed as the intraclass correlation coefficient (ICC) for continuous screening scores and as Cohen’s kappa for categorical agreement; screening completion and compliance rates; referral or escalation rates; recognition and documentation rates; and survey or qualitative accounts of the determinants of nursing practice.

Where screening performance was reported, it was interpreted alongside the tool used, the reference standard applied, the patient population enrolled, and the professional who performed the assessment, because all four shape the reported figures. Implementation studies were charted for tool completion, scoring accuracy, documentation, referral, communication, nurses’ knowledge, attitudes and role clarity, workload, and the extent and type of organizational support. No pooled diagnostic-accuracy estimate or meta-analysis was attempted, because the included studies differed in screening tool, reference standard, clinical setting, population, and method.

### 3.7. Critical Appraisal

In keeping with the scoping design, no formal risk-of-bias assessment or quality appraisal was undertaken. The purpose of the review was to describe the breadth of the literature, characterize the screening methods and implementation contexts reported, and identify evidence gaps, rather than to estimate a pooled effect or assign a certainty grade. JBI guidance treats critical appraisal as optional for this kind of evidence-mapping objective [16].

Because appraisal was not carried out, the results should not be read as comparative judgements about the effectiveness of any particular screening tool or strategy. They are presented as a map of the available evidence.

### 3.8. Synthesis

Study characteristics and quantitative findings from the 18 included studies were synthesized descriptively and organized around the review objectives and the measurement domains set out above, following JBI recommendations for the extraction, analysis, and presentation of scoping review results [19]. The organizing domains were screening and early identification, diagnostic performance and measurement, recognition and documentation, screening and referral completion, and the factors affecting implementation.

Implementation findings from the qualitative, survey, observational, and quality-improvement studies were coded inductively. Recurring content was labeled study by study; the labels were grouped into candidate categories, and the categories were then compared across studies and consolidated into the six domains reported in Section 4.8. Strong recurring themes were the usefulness of a tool for case finding, the accuracy of screening and scoring, the screening-to-action process, nurses’ knowledge and attitudes, and role clarity, together with workflow, documentation, communication, and multidisciplinary support. To avoid conflating different kinds of evidence, studies reporting implementation and context were grouped separately from studies reporting screening validation and diagnostic performance.

The two coauthors who produced the first evidence chart also produced the preliminary descriptive and thematic groupings. The other two coauthors checked these groupings against the chart and the full texts and revised them where the fit was poor. All differences of interpretation were resolved by discussion among the four coauthors. Coding was carried out manually without qualitative analysis software, and independent duplicate coding was not undertaken. This is acknowledged as a limitation in Section 5.6.

## 4. Results

### 4.1. Characteristics of Included Studies

The 18 included studies were published between 1999 and 2026 and were conducted in 10 countries: the United Kingdom, Australia, Belgium, Israel, Italy, Norway, Singapore, Germany, the Netherlands, and Spain. Designs included validation and diagnostic-accuracy studies, cross-sectional audits and surveys, retrospective before-and-after studies, quality-improvement and implementation projects, qualitative interview and focus-group studies, and direct clinical observation. Settings were predominantly geriatric, internal medicine, medical or surgical, acute elderly, and community hospital inpatient wards. Sample sizes ranged from 15 nurses in a qualitative study to 2094 older inpatients in a multicenter ward audit. Table 2 summarizes the included evidence.

### 4.2. Screening Instruments and Measurement Approaches

The MNA and MNA-SF family was the most frequently used older-adult-specific approach across the included evidence. The original MNA contains 18 items and functions as a combined screening and assessment instrument, while the MNA-SF is a six-item short form intended for the rapid identification of malnutrition and nutritional risk in older people [7,8]. The Malnutrition Screening Tool (MST) is a two-item instrument based on recent unintentional weight loss and reduced appetite [5]. MUST combines a body mass index score, an unplanned weight loss score, and an acute-disease effect score into an overall risk category [4]. NRS-2002 combines a nutritional status impairment score with a disease-severity score and adds one point for patients aged 70 years or older [6]. SNAQ is a brief hospital screening instrument based on recent weight loss, reduced appetite, and the use of nutritional supplementation or tube feeding [34].

Two earlier studies used locally developed nursing-specific instruments rather than one of these established tools. Pattison et al. [10] evaluated a qualitative screening tool completed independently by a nurse and a dietitian, and McCall and Cotton [9] evaluated a Nursing Nutritional Assessment Tool designed for acute elderly wards. The remaining studies reported on compliance with nutritional screening, the screening and nutritional-care process, documentation, referral, and nurses’ knowledge and attitudes rather than on the properties of a specific instrument.

Subjective Global Assessment (SGA) [35], the ESPEN diagnostic criteria [36], and GLIM [3] appeared in the included evidence as reference standards or diagnostic frameworks rather than as routine nurse-administered screening tools. Because the studies differed in tool, reference test, assessor, population, and outcome, measurement findings are described study by study rather than combined. Table 3 sets out the instruments and frameworks represented in the included evidence.

### 4.3. Standardized Screening Versus Unaided Clinical Judgment

The clearest evidence favoring standardized screening over informal nursing judgement comes from Gasperini et al. [30]. After the MNA was added to the nursing admission assessment for patients aged 65 years and over, recognition of undernutrition rose from 38.6% to 73.1% (*p* < 0.001). During the period in which both approaches were in use, 38.5% of the patients assessed in that study period were classified as undernourished by the MNA but were not identified as undernourished through the nursing diagnosis, and agreement between the two approaches was poor. Unstructured clinical judgement, used on its own, therefore appears to leave a large share of nutritional problems in hospitalized older adults unrecognized.

Earlier validation work is a reminder that introducing a formal tool does not by itself produce accurate or consistent screening. Pattison et al. [10] found that, among older patients who were objectively undernourished, a nurse classified 58% as at nutritional risk, while a dietitian using the same qualitative tool classified 85%, with poor concordance across all five scoring categories. McCall and Cotton [9] reported the same pattern on an acute elderly ward. Test–retest consistency of the Nursing Nutritional Assessment Tool was acceptable, but nurse–dietitian disagreement persisted, and internal consistency and construct validity were not sufficient to support routine use at that stage.

These findings show that standardization cannot stop at the introduction of a screening tool. In the included evidence, accurate case finding depended on a validated instrument [7,8,22], explicit scoring rules and correct anthropometry [11], staff training and competence [24,33], and demonstrated agreement between assessors [10,12]. ESPEN guidance similarly positions screening with a validated tool at admission as the entry point to the nutrition care process rather than as the process itself [2]. Standardized tools improved case finding relative to unaided judgement [30], but the clinical value of any tool depended on how accurately and consistently nurses applied it [9,10,11,12].

### 4.4. Validity, Accuracy, and Inter-Rater Reliability

The strongest nurse-specific criterion validity evidence comes from Wu et al. [22]. Two registered nurses administered the Malnutrition Screening Tool (MST) to 157 hospitalized adults aged 65 years and over who had been identified as at high risk of hospital readmission. Compared with the Subjective Global Assessment (SGA) [35], the MST achieved 94% sensitivity and 89% specificity in this population. The result supports the use of a brief, nurse-administered tool for routine screening and rescreening, but two features of this study limit how far it can be generalized. The cohort was selected for high readmission risk, and screening was performed by two nurses working on a study protocol. These figures should therefore be read as performance in that defined clinical context rather than as an estimate of what general ward nursing staff achieve during routine admission screening.

Trampisch et al. [12] examined inter-rater reliability directly. Routine MNA-SF screening completed by nurses within 24 h of admission was repeated by a dietitian one to three days later in 105 geriatric inpatients with a mean age of 82.4 years. Agreement on the continuous MNA-SF total score was moderate to good (ICC = 0.74), but agreement on the resulting nutritional risk category was only fair (Cohen’s kappa = 0.37). Because it is the category, and not the raw score, that determines whether a patient is referred for further assessment, disagreement at a category boundary has direct clinical consequences.

Cortes et al. [32] provided comparative diagnostic-accuracy data across several instruments. In a prospective study of 248 internal medicine inpatients with a mean age of 75.2 years, MUST, NRS-2002, MST, SNAQ, and MNA-SF were assessed against the ESPEN diagnostic criteria [36], with SGA and GLIM also applied. For the whole sample, MUST showed 80.0% sensitivity and 74.7% specificity. In the subgroup aged over 65 years (*n* = 172), MNA-SF showed 94.4% sensitivity but only 39.0% specificity, meaning that a large proportion of the patients it flagged were not classified as malnourished by the reference standard. One qualification applies to all these figures. Every assessment in this study was performed by a single experienced clinical nurse and nutritionist, so assessor variability was minimal by design. That strengthens internal consistency, but it limits how far the results describe screening performed routinely by a large and mixed ward nursing workforce.

The earlier validation studies by Pattison et al. [10] and McCall and Cotton [9] should be read alongside these results. Both found classification differences between nurses and dietitians using the same instrument, and McCall and Cotton also reported weak psychometric performance of the nursing tool itself. Taken with Trampisch et al. [12], this indicates that who performs the screening, and how well that person has been trained, affects the result as much as the design of the instrument does.

Taken together, nurse-administered screening tools achieved high sensitivity and acceptable specificity under defined study conditions [22,32], while inter-rater variability persisted across two decades of evidence [10,12]. Cortes et al. [32] showed that instruments differed in the balance of sensitivity and specificity within the same inpatient sample, and Trampisch et al. [12] showed that agreement on risk category was weaker than agreement on the continuous score. The practical consequences drawn here, namely, the assessment workload generated by low specificity, the risk of false-negative classification, and the case for training and periodic reliability checks, are inferences of the review team based on the reported measurement findings. They were not themselves measured as outcomes in any included study. Across the accuracy evidence in this review, reported performance is tied to the population enrolled and to the professional who performed the screening, and neither condition matches routine admission screening on a general ward.

### 4.5. Screening Completion, Scoring Fidelity, and Screening-to-Action Gaps

A consistent gap runs through the included studies: completing a nutritional screen is not the same as scoring it correctly, documenting it, communicating it, and acting on it. Frank et al. [11] described this gap in a three-cycle quality-improvement project on care-of-the-elderly wards. The common shortfalls were missing MUST scores, inaccurate body mass index and weight loss scoring, and high-risk results that were not followed by any recorded action. After nurse education and ward-level intervention, documented MUST completion rose from 73% to 97% and dietitian referral rose from 62% to 86%. These figures show that screening processes respond to targeted intervention. They describe how reliably nutritional risk was identified and referred, not whether the patients concerned subsequently did better.

The larger studies show how persistent these gaps are across hospital systems. Vanderwee et al. [21] audited 2094 older inpatients across 140 hospital wards in Belgium and reported a malnutrition prevalence of 31.9% alongside suboptimal use of standardized screening, assessment, protocols, and follow-up nutritional care. Bonetti et al. [27] reported a malnutrition prevalence of 21.4% among 1066 older medical and surgical inpatients, and around 55% of the participating units reported carrying out no nutritional screening at all. Many units also lacked a structured nutritional history, written protocols, or routine intake monitoring.

The most recent implementation evidence suggests that education, audit and feedback, and stakeholder engagement can shift these figures, though only so far. Arnaldos-Esteban et al. [33] applied a structured JBI best-practice implementation process on internal medicine wards and raised compliance with best-practice nutritional screening for patients over 75 years from 22.62% to 48.26%. Even after the intervention, fewer than half of the eligible patients were screened in line with the criteria, and documentation gaps remained. Implementation barriers evidently survive a well-designed quality-improvement program.

Taken together, these studies indicate that screening completion on its own does not describe how the pathway performs. Frank et al. [11] documented missing scores, questionable anthropometry, and unacted-on high-risk results alongside improved documentation and referral after intervention. Vanderwee et al. [21] and Bonetti et al. [27] reported that screening, assessment, and subsequent nutritional care were frequently absent at ward level. Arnaldos-Esteban et al. [33] found that best-practice compliance still reached only about half of eligible patients after a structured implementation strategy, and Adams et al. [20] and Halvorsen et al. [26] described failures of recognition, documentation, and communication occurring after the screening step. On this basis, accurate scoring, documentation, referral, and a defined response to identified risk are treated in this review as components of the nurse-led screening pathway. This grouping is the review team’s interpretive synthesis of the included evidence. No included study evaluated the pathway as a single defined intervention.

### 4.6. Knowledge, Attitudes, Role Clarity, and Professional Ownership

Nurses’ knowledge, attitudes, perceived responsibility, and sense of ownership emerged repeatedly as determinants of whether nutritional screening and care actually happen. Boaz et al. [24] surveyed 106 hospital nurses and found a mean nutrition-knowledge score of 51.9% correct. Nutritional assessment and feeding-related tasks were also ranked low against other clinical responsibilities, which points to a gap in perceived responsibility as much as in knowledge.

Bonetti et al. [28] surveyed 799 nurses working in medical and surgical units across 10 hospitals and found considerable variation in attitudes toward the nutritional care of older people: 23.2% held negative attitudes, 56.6% neutral attitudes, and only 20.2% positive attitudes. The authors linked these attitudes to education, perceived professional responsibility, and the level of organizational support available on the unit. Qualitative studies point in the same direction. Eide et al. [25] identified professional isolation, competence needs, an inflexible food service, system failure, and the low priority given to nutrition as the main barriers reported by hospital nurses. Neo et al. [29] found that enrolled nurses valued bedside observation but were constrained by workload, role ambiguity, limited resources, and competing priorities. Bonetti et al. [23] observed that visible nutritional problems at mealtimes were not consistently prioritized and attributed this to competing tasks and limited ownership of the problem. Read together, these findings indicate that implementing nurse-led screening is not only a matter of selecting a tool. It also depends on whether nurses regard screening as part of their professional role, understand why it matters, and have the organizational support needed to act on the result.

### 4.7. Documentation, Communication, and Multidisciplinary Integration

The benefit of early identification can be lost if the finding is poorly documented or never reaches the rest of the team. Halvorsen et al. [26] described unsystematic and fragmentary nutrition documentation, incomplete recording at admission, and discontinuous transfer of nutrition information between settings, all of which weaken the link between an initial finding and any subsequent assessment or intervention. Van den Berg et al. [31] observed nurses directly over 183 h and found that screening and assessment took place within a wider clinical process shaped by workflow, ownership, communication, knowledge, and multidisciplinary collaboration. Nutritional care in practice was not a self-contained nursing task but part of a shared process of assessment, documentation, planning, monitoring, and communication, in which patient participation was uncommon.

Adams et al. [20] and Bonetti et al. [27] add evidence from the steps that follow screening. Adams et al. found that weight loss and reduced appetite were rarely recognized or documented and that dietetic referral was uncommon, while Bonetti et al. reported an absence of structured nutritional histories, screening, assessment, and monitoring at unit level. Together, these findings support treating nurse-led nutritional screening as a multistep clinical pathway rather than a stand-alone tool. The pathway runs from accurate screening through documentation of nutritional risk, communication at handover, reassessment where indicated, referral, and linkage to a specific nutritional care plan.

### 4.8. Cross-Study Synthesis of Barriers and Facilitators

Grouping the barriers and facilitators reported across the 18 included studies produced six interrelated domains: tool and measurement; knowledge and confidence; role clarity and professional ownership; workflow and resources; documentation and communication; and the screening-to-action pathway. These domains describe the barriers and facilitators observed across different study types and contexts, including validation, observational, qualitative, survey, and implementation designs. They do not represent the results of a single standardized intervention evaluated across studies. Table 4 presents the domains with illustrative evidence.

## 5. Discussion

### 5.1. Principal Findings

This scoping review mapped 18 studies identified through MEDLINE via PubMed, Scopus, and Web of Science Core Collection, spanning more than two decades of research on nurse-led nutritional screening and early identification among hospitalized older adults. Four conclusions follow from the mapped evidence. First, structured screening identifies nutritional risk that unaided nursing judgement misses. Second, nurse-administered tools can show good criterion validity in selected populations, while agreement between assessors on the resulting risk category remains inconsistent. Third, the largest practice deficits cluster around and after the act of screening: inaccurate measurement, incomplete documentation, missed referrals, and absent follow-up. Fourth, implementation is shaped by nurses’ knowledge, attitudes, workload, and role clarity, and by the design of the multidisciplinary pathway.

These findings caution against treating a screening tool as a solution. Gasperini et al. [30] showed a large increase in recognition once the MNA was built into the nursing admission assessment, but Pattison et al. [10], McCall and Cotton [9], and Trampisch et al. [12] all showed that assessor disagreement survives the introduction of a structured instrument. Frank et al. [11] added that a completed screen may be incorrectly scored and that a high score may not trigger anything at all. A high-quality nurse-led program therefore requires a validated instrument, accurate measurement, trained users, standardized timing, interpretable thresholds, and a closed-loop response pathway.

The improvements mapped in this review are process and case-finding improvements. The rise in recognized undernutrition from 38.6% to 73.1% after the MNA was added to the nursing admission assessment [30], the increase in documented MUST completion from 73% to 97% and in dietitian referral from 62% to 86% [11], and the compliance gains reported by Arnaldos-Esteban et al. [33] all describe how reliably nutritional risk is detected and acted on. None of the included studies measured whether these changes were followed by fewer complications, shorter length of stay, fewer readmissions, less functional decline, or lower mortality, and none used a design capable of attributing such changes to screening.

### 5.2. Interpreting Tool Performance in Older Inpatients

No single instrument emerged as universally superior. The MNA and MNA-SF were designed for older adults and are highly sensitive in this population, which is desirable when the priority is to avoid missed cases. The low specificity of the MNA-SF in the subgroup aged over 65 years of Cortes et al. [32] means, however, that many patients flagged as at risk will need further assessment before anything is concluded. MST offers a shorter format and showed strong sensitivity and specificity in the older, high-readmission-risk cohort of Wu et al. [22]. That estimate came from a selected population screened by two nurses working to a study protocol, and it should not be extended to unselected older inpatients screened by general ward nursing staff. The same caution applies to the figures from Cortes et al. [32], where all assessments were made by a single experienced clinical nurse and nutritionist. Across the accuracy evidence in this review, reported performance is tied to the population enrolled and to who performed the screening, and neither condition matches routine admission screening on a general ward. The accuracy figures should therefore be read as what these instruments can achieve in defined populations under favorable assessment conditions, not as what a hospital should expect from its own ward-based screening. MUST is practical and linked to an action framework, but its dependence on body mass index and previous weight makes it vulnerable when weights are unavailable or estimated [11]. NRS-2002 integrates disease severity and older age explicitly, at the cost of requiring more clinical judgement than the very brief tools.

These differences reinforce the distinction between screening and diagnosis. A nurse screening tool should optimize case finding and trigger an appropriate next step; it does not deliver a diagnosis of malnutrition. GLIM places screening explicitly before diagnostic assessment [3], and the ESPEN diagnostic criteria likewise require assessment beyond a screening score [36]. As additional clinical context outside the 18-study evidence map, a 2025 cross-sectional study of hospitalized older adults reported differences in routine biochemical, hematological, and inflammatory parameters across nutritional status groups defined by the MNA and by the Geriatric Nutritional Risk Index [37]. That work supports broader assessment after a positive screen, but it does not convert a screening classification into a diagnosis. High sensitivity may therefore be clinically acceptable where the service has sufficient capacity for confirmatory assessment, while settings with limited dietetic resources may need to balance sensitivity against specificity.

### 5.3. Implications for Nursing Practice

The recommendations in this section are implications for the screening and care pathway. They should not be read as evidence that nurse-led screening on its own improves clinical outcomes, because no included study measured complications, length of stay, readmission, functional decline, or mortality after screening was introduced. The pathway proposed here is an interpretive synthesis of the included evidence rather than a pathway that has been evaluated as a single intervention. ESPEN guidance recommends routine screening of older people with a validated tool at hospital admission [2]. In the included studies, structured nurse-led screening improved recognition [30], while routine practice was affected by scoring errors and by nurse–dietitian or inter-rater disagreement [9,10,11,12]. Taken together, these findings support the use of a validated, locally approved instrument, completion within a clearly defined institutional timeframe, and training in the measurements that generate the score, including body weight, recent weight loss, appetite or intake change, and the handling of missing data. Periodic competency and reliability checks are proposed because knowledge gaps, scoring inaccuracies, and variation between assessors were reported across the included evidence [11,12,24,33].

The proposed screening-to-action pathway also rests on studies reporting poor recognition and documentation, incomplete protocol use, unacted-on high-risk scores, low referral rates, and fragmented communication [11,20,21,26,27,31,33]. Consistent with the GLIM screening–assessment framework [3], a positive screen should trigger further assessment and a set of predefined actions: documentation of risk, escalation, dietitian referral, initiation of an appropriate nutrition care plan, rescreening, and communication at transfer or discharge. Audit should examine more than completion. The suggested indicators are the proportion of eligible patients screened within the institutional timeframe; the proportion with complete and correct scores or valid anthropometry; periodic inter-rater agreement or error rates; the proportion of positive screens referred; time to dietetic assessment; the proportion with a documented nutrition plan; and rescreening where indicated. These are review-derived quality indicators intended to address the pathway failures mapped in the included studies. They are not a formally validated core indicator set.

### 5.4. Implications for Hospital Policy and Quality Improvement

Hospital policy should not treat screening completion as the sole indicator of effective implementation. Frank et al. [11] found that a recorded MUST screen could contain an incorrect body mass index or weight loss score, and that high-risk results did not always lead to the recommended action. Vanderwee et al. [21] and Bonetti et al. [27] likewise reported that screening, protocol use, and subsequent nutritional-care practices did not consistently occur together. Arnaldos-Esteban et al. [33] improved compliance through a structured implementation project, but documentation gaps remained afterwards. This is the specific reason for looking beyond completion: in the included evidence, completion, scoring accuracy, protocol adherence, and referral behave as separate aspects of performance and do not move together, so a completion-only indicator can overstate performance because a recorded screen may be inaccurate, incompletely documented, or disconnected from referral and care. Hospital policy should therefore monitor completion alongside score correctness, protocol adherence, documentation, and the action taken after a positive screen [11,21,27,33]. Electronic prompts may support this pathway, but that remains a review-derived implementation proposal rather than a strategy directly evaluated in the included studies. It should also be clear that every indicator proposed here is a process indicator. Each describes how completely and how correctly nutritional risk is identified, documented, and acted on. Because the included studies did not follow patients through to complications, length of stay, readmission, function, or mortality, better performance on these indicators can be presented as better case finding and a better-functioning care pathway, but not as demonstrated clinical benefit.

Multidisciplinary governance matters here as well, because the included qualitative studies identified professional isolation, role ambiguity, and fragmented communication as barriers to nutritional care [25,26,29,31]. Nurses are well placed to initiate screening, while diagnostic assessment and individualized treatment usually require dietetic and medical input, which is consistent with the screening-to-assessment distinction drawn by GLIM [3]. The implementation evidence supports several specific strategies. Frank et al. [11] combined repeated audit cycles, staff education, and weekly dietitian ward rounds, and observed improvements in documented screening and dietitian referral. Arnaldos-Esteban et al. [33] used a structured JBI audit-and-feedback process with barrier analysis, targeted training, stakeholder engagement, and a follow-up audit, and reported improved compliance alongside better nurse-reported knowledge and confidence. Both are uncontrolled quality-improvement studies, so they support repeated audit and feedback, focused training, stakeholder engagement, and scheduled dietitian involvement as reasonable implementation options without establishing the independent effectiveness of any single component. Ward-level champions and feedback on referral completion were not evaluated as discrete strategies in any included study and are therefore not presented here as evidence-based recommendations.

### 5.5. Research Gaps

Several gaps limit the maturity of this evidence base. First, few studies compare nurse-administered screening against an independent, currently accepted comparator such as dietitian-administered SGA [35] or a malnutrition diagnosis made with the ESPEN or GLIM criteria [3,36]. In this review, a reference standard means the independent assessment used to decide whether a screening result has correctly identified malnutrition or nutritional risk, and contemporary means that this comparator reflects current consensus diagnostic criteria, most commonly GLIM or the ESPEN criteria, and is applied by an appropriately trained assessor working independently of the nurse who performed the screen. Second, validation studies tend to report sensitivity and specificity without reporting whether screening led to timely assessment, treatment, or improved outcomes. Third, implementation research commonly reports screening completion without quantifying scoring accuracy or downstream referral fidelity. Fourth, few multicenter studies evaluate the same nurse-led pathway across hospitals, which limits generalizability. Fifth, evidence remains sparse on rescreening during prolonged admissions, transitions between wards, digital decision support, and the effect of cognitive impairment, delirium, edema, immobility, or the inability to obtain a standing weight on tool performance.

Future studies should prospectively report the denominator of eligible older inpatients, screening timing, the professional role of the screener, how missing data were handled, the tool thresholds applied, the reference standard used, sensitivity and specificity or agreement statistics where appropriate, referral and treatment initiation, rescreening, and patient outcomes such as length of stay, complications, readmission, function, and mortality. Hybrid effectiveness-implementation designs appear particularly well suited to this question, since the main challenge is not identifying a valid tool but embedding one reliably in routine clinical care.

### 5.6. Strengths and Limitations

This review has several strengths. Three bibliographic databases were searched using explicit PCC criteria, and direct screening-performance evidence was kept separate from implementation and context evidence throughout the synthesis. A strict practicing-nurse criterion was applied, and studies in which the nursing role in screening or early identification was not explicit were excluded rather than assumed. Measurement findings were charted within defined domains rather than combined across incompatible frameworks, constructs, and designs. The review also reports the full database-level search pathway, retrieval and deduplication counts, search dates, and the reviewer workflow applied at each stage, so that the process can be appraised and repeated.

Several limitations should be read alongside these strengths. The rapid approach meant that CINAHL, Embase, the Cochrane Library, trial registries, dedicated grey literature sources, and dissertation repositories were not searched, so relevant studies indexed only in those sources may have been missed. No formal backward or forward citation searching was carried out; the verification step confirmed indexing and bibliographic accuracy and added no records to the evidence set. The July verification did not update the June 2026 database searches, so studies first made available in the searched databases after the respective search dates may have been missed.

The database strategies reported in Appendix A were reconstructed from the saved searches rather than recorded verbatim at the time of execution, which is a reporting limitation even though retrieval counts, deduplication totals, and the months of search execution and verification are reported. Screening and chart review followed a sequential review-and-revision process involving four coauthors rather than fully independent duplicate screening; therefore, no inter-reviewer agreement statistics could be calculated, and inductive coding was not independently duplicated. Record-level reasons were not retained for the 1147 records excluded at title and abstract screening. Restriction to English-language full texts introduces a further limitation. Finally, no formal methodological quality or risk-of-bias assessment was conducted. That was appropriate for an evidence-mapping objective, but it means the findings should be interpreted as a map of the available evidence rather than as graded evidence of effectiveness.

## 6. Conclusions

Nurse-led nutritional screening can improve the early identification of malnutrition risk among hospitalized older adults, particularly where standardized tools replace unaided clinical judgement. The available evidence supports the feasibility of the MNA and MNA-SF, MST, MUST, and related approaches within nursing workflows, and it also shows that performance depends on accurate scoring, staff competence, and the reference standard used for comparison. This conclusion is limited to case finding and to the screening process itself. The included studies reported better recognition, higher screening completion, and more dietitian referrals; they did not assess whether nurse-led screening reduces complications, length of stay, readmission, functional decline, or mortality, and no claim of clinical benefit can be drawn from this evidence base. The central implementation problem is not simply whether nurses screen. It is whether the screen is correct, documented, communicated, and linked to timely assessment and intervention. Hospitals should therefore evaluate the whole screening-to-action pathway rather than the completion rate alone. Future multicenter studies should combine nurse-specific measurement performance with implementation fidelity and patient-centered outcomes, so that the link between better identification and better patient outcomes can be tested directly rather than assumed.

## 7. Implications for Practice

The points below are implications for nutritional screening and for the care pathway that follows a positive screen. They are drawn from evidence on recognition, scoring accuracy, documentation, and referral. The included studies did not evaluate patient outcomes, so these points should not be read as evidence that nurse-led screening reduces complications, length of stay, readmission, functional decline, or mortality.

- Use a validated screening instrument appropriate to the older inpatient population rather than relying on unaided clinical judgement alone [2,22,30].
- Define an institutional screening window beginning at hospital admission, together with a rescreening policy for prolonged admission or clinical deterioration. ESPEN guidance recommends screening with a validated tool at admission and repeated screening during the hospital stay [2], and the included implementation studies show that screening and rescreening do not occur reliably unless the timing is defined and audited [11,33].
- Provide nurses with competency-based training in the measurements that generate the score, including body weight, body mass index, recent weight loss, appetite or intake change, and the handling of missing data. This matters because inaccurate or estimated measurements and inconsistent scoring can misclassify nutritional risk and prevent appropriate referral or care [9,10,11,12,24,33].
- Audit both completion and correctness, including a periodic inter-rater reliability check [11,12,21,27,33].
- Create explicit escalation pathways for high-risk results, including further assessment, dietitian referral, and documented nutrition care plans [3,11,20,21,33].
- Treat documentation, handover, and multidisciplinary communication as part of the screening intervention rather than as separate administrative tasks [26,31].

## Figures and Tables

**Table 1:** Population–Concept–Context eligibility criteria.

PCC/Criterion	Included	Excluded
Population	Hospitalized adults 65 years of age or older, or populations described as older, elderly, or geriatric. Studies with mixed age populations were accepted only if results for older adults could be extracted separately.	All-age cohorts without separately extractable older-adult data; pediatric populations; younger-adult populations.
Concept	Studies that analyze how practicing nurses engaged in nutritional screening, early recognition or identification of malnutrition or nutritional risk, case finding related to nutrition, documentation or communication following identification, referral or escalation of positive screens, or determinants directly related to the implementation of these processes.	Screening undertaken only by dietitians, physicians, research staff, patients, or nursing students; studies in which the nursing role in screening or early identification was not explicit; treatment-only or nutritional-support interventions conducted after malnutrition or nutritional risk had already been identified.
Context	Acute or subacute inpatient hospital services like medical, surgical, geriatric, internal medicine, and specialty community hospital units.	Community, outpatient, home-care, nursing-home/long-term-care, rehabilitation-only, and emergency-department-only settings unless eligible inpatient findings were separately extractable.
Evidence type	Peer-reviewed primary empirical research containing quantitative, qualitative, mixed-methods, validation, diagnostic-accuracy, observational, cross-sectional, quality-improvement, and implementation research.	Reviews, narrative reviews, guidelines and editorials, commentaries, case reports, dissertations/theses, and abstracts that do not feature a complete empirical report.
Language	Full-text articles available in English.	Non-English full texts when a reliable English version or translation was not available.
Date	Studies published from 1 January 1999 to 31 July 2026 were retrieved in the June 2026 database searches. This publication-date window did not extend search coverage beyond the respective June 2026 search dates. No separate allowance was made for earlier foundational studies.	Studies published before 1 January 1999 or after 31 July 2026.
Duplicate/overlapping reports	Reports provide distinct eligible data or outcomes.	Duplicate records and overlapping reports without distinct eligible findings.

**Table 2:** Characteristics and principal findings of included studies.

Study	Country/Setting	Design/Sample	Screening or Identification Method	Nurse-Led Component	Key Findings
Pattison et al. (1999) [10]	United Kingdom; elderly hospital population; St John’s Hospital	Validation study; *n* = 66; mean age 78.5 years	Local qualitative nutritional screening tool vs. physical markers	Nurse and dietitian independently applied the same tool	Undernutrition prevalence 23%; dietitian classified 85% of undernourished patients as at risk versus 58% by the nurse; no agreement across the five scoring categories.
McCall & Cotton (2001) [9]	United Kingdom; Acute elderly ward	Validation study; 185 consecutive admissions	Nursing Nutritional Assessment Tool	Two nurses and a dietitian tested the tool	Test–retest consistency was acceptable, but nurse–dietitian inter-rater disagreement was persistent; internal consistency and construct validity were insufficient for routine use at that stage.
Adams et al. (2008) [20]	Australia; medical wards, tertiary teaching hospital	Quantitative/qualitative recognition study; 100 elderly inpatients + 57 health professionals	MNA plus staff questionnaire and chart recognition/referral review	Nursing recognition and documentation were explicitly evaluated	30% were malnourished and 61% at risk; weight loss and appetite were poorly recognized/documented, and dietetic referral was uncommon.
Vanderwee et al. (2011) [21]	Belgium; 140 hospital wards for older people	Multicenter cross-sectional audit; *n* = 2094 older inpatients	Ward screening and nutritional-care practice audit	Ward nursing practices/protocols were central to data collection	Malnutrition prevalence was 31.9%; systematic screening, assessment, protocol use, and nutritional-care practices were suboptimal.
Wu et al. (2012) [22]	Australia; acute hospital; patients at high risk of readmission	Criterion validity study; *n* = 157; age ≥ 65 years	MST vs. SGA	Two registered nurses administered the MST	MST sensitivity 94% and specificity 89% versus SGA in a cohort selected for high readmission risk and screened by two nurses working to a study protocol.
Bonetti et al. (2013) [23]	Italy; hospital wards caring for older people	Observational nursing-perception study; Hospital nurses caring for older inpatients	Mealtime observation and nurses’ perception of malnutrition	Direct examination of nurses’ recognition and response	Visible nutritional problems were not consistently prioritized; competing tasks and limited ownership contributed to under-recognition.
Boaz et al. (2013) [24]	Israel; two government hospitals	Cross-sectional nurse survey; *n* = 106 nurses	Knowledge/attitude and clinical-task questionnaire	Direct assessment of nurses’ nutrition knowledge and attitudes	Mean correct nutrition-knowledge score was 51.9%; nutrition assessment and feeding-related tasks were ranked relatively low, indicating educational needs.
Eide et al. (2015) [25]	Norway; seven somatic hospital wards	Qualitative study; *n* = 16 hospital nurses	Interviews on nutritional care of undernourished older patients	Practicing nurses described assessment and care decisions	Five barrier themes included professional isolation, competence needs, inflexible food service, system failure, and low prioritization of nutrition.
Frank et al. (2015) [11]	United Kingdom; care-of-the-elderly wards, Watford General Hospital	Quality-improvement audit (three cycles); *n* = 64, 62, and 63 ward patients across audit cycles	MUST documented in nursing notes; recalculation and intervention audit	Nursing documentation and scoring were the primary implementation target	MUST completion increased from 73% to 97%; dietitian referrals increased from 62% to 86% in the second audit cycle. Incorrect BMI/weight loss scoring and failure to act on high scores were recurrent problems.
Halvorsen et al. (2016) [26]	Norway; Hospitals and nursing homes; hospital findings extracted	Qualitative focus groups; 16 hospital nurses; additional non-hospital participants not synthesized	Documentation and communication of nutritional care	Hospital nurses described admission screening/documentation and information transfer	Documentation was unsystematic and nutrition information transfer fragmented, weakening continuity from identification to care.
Bonetti et al. (2017) [27]	Italy; 12 hospitals, 80 medical/surgical units	Multicenter cross-sectional study; *n* = 1066 older inpatients	Full MNA plus unit-level nutritional-care assessment	Head nurses supplied unit-practice data	228 patients (21.4%) were malnourished; 55% of units reported no nutrition screening, and many lacked structured nutritional history, protocols, or intake monitoring.
Bonetti et al. (2021) [28]	Italy; Medical and surgical units in 10 hospitals	Cross-sectional nurse-attitude study; 799 nurses (61.8% response)	Staff Attitudes to Nutritional Nursing Care Geriatric scale	Direct measurement of nurses’ attitudes	23.2% had negative, 56.6% neutral, and 20.2% positive attitudes; education, responsibility, and organizational support were implicated in practice quality.
Neo et al. (2021) [29]	Singapore; Acute-care hospital	Qualitative descriptive study; *n* = 15 enrolled nurses	Interviews on nutritional assessment and care	Nurses described bedside identification and care delivery	Bedside observation was valued, but workload, role ambiguity, resources, and competing priorities constrained systematic nutritional care.
Gasperini et al. (2022) [30]	Italy; Community hospital	Retrospective before-and-after study; ~316 patients age ≥ 65 (238 before; 78 after)	MNA added to nursing admission assessment	Nurses completed MNA and documented nursing diagnoses	Recognition of undernutrition increased from 38.6% before MNA implementation to 73.1% after implementation. Among patients assessed during the 2020 study period, 38.5% were identified as undernourished by MNA but not by the nursing diagnosis.
Trampisch et al. (2022) [12]	Germany; Geriatric hospital	Retrospective cross-sectional inter-rater study; *n* = 105; mean age 82.4 years	MNA-SF	Routine nurse MNA-SF within 24 h; dietitian repeated 1–3 days later	ICC for the total score was 0.74, but categorical agreement was only fair (Cohen’s kappa = 0.37), indicating clinically relevant misclassification at category boundaries.
van den Berg et al. (2023) [31]	Netherlands; Medical wards including a geriatric ward	Qualitative observational study + focus groups; 39 nurses observed for 183 h; 24 nurses on geriatric ward	Observed screening, assessment, care planning, monitoring, participation	Direct observation of nurses delivering nutritional care	Screening occurred, but practice was shaped by workflow, ownership, communication, knowledge, and multidisciplinary collaboration; patient participation was uncommon.
Cortes et al. (2024) [32]	Spain; Internal medicine wards	Prospective cross-sectional diagnostic-accuracy study; *n* = 248; mean age 75.2; >65 subgroup *n* = 172	MUST, NRS-2002, MST, SNAQ, MNA-SF vs. ESPEN; SGA/GLIM also assessed	One experienced clinical nurse/nutritionist performed all evaluations	Overall, MUST sensitivity was 80.0% and specificity 74.7% against the ESPEN criteria; among patients aged over 65, MNA-SF sensitivity was 94.4% but specificity 39.0%. All assessments were performed by one experienced clinical nurse and nutritionist.
Arnaldos-Esteban et al. (2026) [33]	Spain; Internal medicine wards	JBI best-practice implementation project; Patients > 75 years in baseline/follow-up audit periods	Best-practice nutritional screening criteria with audit/feedback	Nurses central to screening implementation; education/stakeholder strategies applied	Compliance with best-practice nutritional screening increased from 22.62% to 48.26%; nurses reported improved knowledge/confidence, although documentation gaps remained.

**Table 3:** Screening and measurement approaches represented in the included evidence.

Instrument/Framework	Core Content/Thresholds	Use in Included Evidence	Relevant Measurements
MNA (full)	18-item geriatric nutritional assessment; total score 0–30. Conventional categories: <17 malnourished; 17–23.5 at risk; ≥24 normal nutritional status.	Gasperini et al. (2022) [30]; Adams et al. (2008) [20]; Bonetti et al. (2017) [27]. Foundational instrument: Vellas et al. (1999) [8].	Nutritional status classification, prevalence, recognition, and comparison with nursing judgement or routine care.
MNA-SF	Six-item short form; total score 0–14. Conventional categories: 0–7 malnourished; 8–11 at risk; 12–14 normal nutritional status.	Trampisch et al. (2022) [12]; Cortes et al. (2024) [32]. Foundational instrument: Rubenstein et al. (2001) [7].	Inter-rater reliability, ICC, Cohen’s kappa, sensitivity, specificity, and categorical classification.
MST	Two-item screening tool based on recent unintentional weight loss and reduced appetite; score ≥ 2 commonly indicates nutritional risk.	Wu et al. (2012) [22]; Cortes et al. (2024) [32]. Foundational instrument: Ferguson et al. (1999) [5].	Sensitivity, specificity, criterion validity, agreement with SGA, and risk classification.
MUST	BMI score + unplanned weight loss score + acute-disease effect score. Overall risk classification: 0 low risk; 1 medium risk; ≥2 high risk.	Frank et al. (2015) [11]; Cortes et al. (2024) [32]. Foundational framework: BAPEN (2003) [4].	Screening completion, scoring accuracy, referral, sensitivity, specificity, and risk classification.
NRS-2002	Nutritional status impairment (0–3) + disease severity (0–3), with an additional 1 point for age ≥70 years; total score ≥3 indicates nutritional risk.	Cortes et al. (2024) [32]. Foundational instrument: Kondrup et al. (2003) [6].	Diagnostic accuracy and nutritional risk classification.
SNAQ	Brief hospital screening instrument incorporating recent weight loss, reduced appetite, and use of nutritional supplementation/support.	Cortes et al. (2024) [32]. Foundational instrument: Kruizenga et al. (2005) [34].	Diagnostic accuracy and comparative screening performance.
Local qualitative nutritional screening tool	Locally developed qualitative assessment incorporating clinical indicators of nutritional risk; evaluated against physical markers.	Pattison et al. (1999) [10].	Nurse–dietitian agreement, case recognition, and comparison with objective markers of undernutrition.
Nursing Nutritional Assessment Tool	Nursing-specific assessment instrument developed for use with older adults admitted to acute elderly wards.	McCall & Cotton (2001) [9].	Test–retest consistency, nurse–dietitian inter-rater agreement, internal consistency, and construct validity.
Reference/diagnostic frameworks	SGA, the ESPEN diagnostic criteria, and GLIM were applied as reference standards or diagnostic frameworks rather than as routine nurse-administered screening tools.	Wu et al. (2012) [22]; Cortes et al. (2024) [32]. SGA: Detsky et al. (1987) [35]; ESPEN diagnostic criteria: Cederholm et al. (2015) [36]; GLIM framework: Cederholm et al. (2019) [3].	Criterion validity, sensitivity/specificity comparisons, and diagnostic agreement.

**Table 4:** Cross-study synthesis of barriers and facilitators across the included evidence.

Domain	Recurring Barriers	Facilitators/Practical Strategies	Illustrative Evidence
Tool and measurement	Inconsistent scoring; disagreement between assessors; boundary-category misclassification; inaccurate BMI or weight loss estimates; variation in specificity between tools.	Use of validated tools appropriate for older adults; clear scoring instructions; staff competency assessment; periodic inter-rater reliability checks.	Pattison et al. (1999) [10]; McCall & Cotton (2001) [9]; Frank et al. (2015) [11]; Trampisch et al. (2022) [12]; Cortes et al. (2024) [32]
Knowledge and confidence	Gaps in nutrition knowledge; low perceived priority of nutrition; reliance on informal clinical judgement; limited confidence in screening procedures.	Targeted education; practical or case-based training; audit and feedback; accessible screening protocols and guidance.	Boaz et al. (2013) [24]; Bonetti et al. (2021) [28]; Gasperini et al. (2022) [30]; Arnaldos-Esteban et al. (2026) [33]
Role clarity and professional ownership	Unclear responsibility for nutritional screening and care; role ambiguity; professional isolation; limited ownership of nutritional problems.	Explicit definition of nursing responsibility for screening; clear escalation responsibilities; stronger integration with dietitians and other professionals.	Bonetti et al. (2013) [23]; Eide et al. (2015) [25]; Neo et al. (2021) [29]; van den Berg et al. (2023) [31]
Workflow and resources	Competing clinical priorities; workload and staffing pressures; lack of time; nutrition deprioritized within routine care.	Integration of screening into admission workflow; clearly defined timing; availability of necessary measurements; planned rescreening when clinically indicated.	Bonetti et al. (2013) [23]; Frank et al. (2015) [11]; Neo et al. (2021) [29]; van den Berg et al. (2023) [31]
Documentation and communication	Incomplete nutritional histories; fragmented records; poor transfer of nutrition information; inconsistent documentation of risk and care plans.	Structured documentation fields; standardized handover processes; clearly visible nutritional risk status; consistent communication across disciplines.	Adams et al. (2008) [20]; Halvorsen et al. (2016) [26]; Bonetti et al. (2017) [27]; van den Berg et al. (2023) [31]
Screening-to-action pathway	Positive or high-risk scores not acted upon; inadequate referral; inconsistent protocol use; weak follow-up after identification.	Clear escalation and referral criteria; dietitian collaboration; audit and feedback; linkage of positive screening results to assessment and nutritional care planning.	Vanderwee et al. (2011) [21]; Frank et al. (2015) [11]; Bonetti et al. (2017) [27]; Arnaldos-Esteban et al. (2026) [33]

## Data Availability

The study-level evidence chart for the 18 included studies is available from the corresponding author upon reasonable request. The chart includes bibliographic information, study characteristics, screening or early-identification approaches, nurse-led components, and principal findings of the included studies. The full database search strategies, retrieval counts, deduplication totals, and search dates are reported in Section 3.3 and Appendix A.

## Appendix A. Search Concepts and Evidence-Set Audit Notes

Three bibliographic databases were searched: MEDLINE via PubMed, Scopus, and Web of Science Core Collection. The search strategy was developed around five principal concept blocks corresponding to the review question and the PCC framework: older adults, the inpatient hospital setting, malnutrition or nutritional risk, nursing, and screening or early identification. Synonymous terms within each concept block were combined using OR, and the principal blocks were combined using AND. Database-specific controlled vocabulary, field codes, and syntax were used where appropriate. The publication-date eligibility window was 1 January 1999 to 31 July 2026. All database searches were executed in June 2026, and search coverage was limited to records available on the respective search dates. The final evidence set underwent bibliographic and indexing verification in July 2026; this did not involve rerunning or updating the database searches and did not extend search coverage to 31 July 2026.

**Table A1:** Search concepts and example terms.

Concept	Example Terms
Population	older adult OR older patient OR elderly OR geriatric OR aged
Context	hospital OR inpatient OR acute care OR medical ward OR surgical ward OR geriatric ward OR internal medicine
Nursing role	nurse OR nursing OR nurse-led OR nurse administered OR nursing staff OR registered nurse
Condition	malnutrition OR undernutrition OR nutritional risk OR nutrition risk
Identification process	screen OR identify OR recogn OR assess OR case finding OR admission assessment OR document OR referral OR nutrition care OR barrier OR facilitator OR implement
Tool-specific terms	MNA OR MNA-SF OR Mini Nutritional Assessment OR MST OR Malnutrition Screening Tool OR MUST OR Malnutrition Universal Screening Tool OR NRS-2002 OR SNAQ

### Appendix A.1. Medline via PubMed

The PubMed strategy combined Medical Subject Headings (MeSH) and free-text terms relating to older adults, hospitalization, malnutrition, nursing, and nutritional screening or early identification. The search was conducted in June 2026 and retrieved 793 records. The reported publication-date limits were 1 January 1999 to 31 July 2026; these limits did not extend retrieval beyond records available on the June 2026 search date. The strategy, reconstructed from the saved search, was:

(“Aged”[Mesh] OR aged[Title/Abstract] OR elderly[Title/Abstract] OR elder*[Title/Abstract] OR “older adult*”[Title/Abstract] OR “older patient*”[Title/Abstract] OR geriatric*[Title/Abstract])
AND (“Hospitals”[Mesh] OR hospital*[Title/Abstract] OR inpatient*[Title/Abstract] OR “acute care”[Title/Abstract] OR “medical ward*”[Title/Abstract] OR “surgical ward*”[Title/Abstract])
AND (“Malnutrition”[Mesh] OR malnutrition[Title/Abstract] OR undernutrition[Title/Abstract] OR “nutritional risk”[Title/Abstract] OR “nutrition risk”[Title/Abstract] OR “protein-energy malnutrition”[Title/Abstract]) AND (“Nurses”[Mesh] OR nurse*[Title/Abstract] OR nursing[Title/Abstract] OR “nurse-led”[Title/Abstract] OR “nurse administered”[Title/Abstract]) AND (“Nutrition Assessment”[Mesh] OR screen*[Title/Abstract] OR identif*[Title/Abstract] OR recogn*[Title/Abstract] OR assess*[Title/Abstract] OR “case finding”[Title/Abstract] OR document*[Title/Abstract] OR referral[Title/Abstract] OR “nutrition care”[Title/Abstract] OR barrier*[Title/Abstract] OR facilitator*[Title/Abstract] OR implement*[Title/Abstract] OR MNA[Title/Abstract] OR MNA-SF[Title/Abstract] OR MST[Title/Abstract] OR MUST[Title/Abstract] OR NRS-2002[Title/Abstract] OR SNAQ[Title/Abstract])

### Appendix A.2. Scopus

The Scopus search was conducted in the TITLE-ABS-KEY fields and retrieved 550 records. The strategy was:

TITLE-ABS-KEY ((“older adult*” OR “older patient*” OR elderly OR geriatric) AND (hospital OR inpatient OR “acute care” OR “medical ward” OR “surgical ward*”) AND (malnutrition OR undernutrition OR “nutritional risk” OR “nutrition risk”) AND (nurse* OR nursing)
AND (screen* OR identify* OR recogn* OR “case finding” OR document* OR referral OR “nutrition care” OR barrier* OR facilitator* OR implement)
AND PUBYEAR AFT 1998
AND PUBYEAR BEF 2027

### Appendix A.3. Web of Science Core Collection

The Web of Science Core Collection search used the Topic field and retrieved 400 records. The strategy was as follows:

(“older adult*” OR “older patient*” OR elderly OR geriatric) AND (hospital* OR inpatient* OR “acute care” OR “medical ward*” OR “surgical ward”) AND (malnutrition OR undernutrition OR “nutritional risk” OR “nutrition risk”) AND (nurse* OR nursing) AND (screen OR identif* OR recogn OR “case finding” OR document* OR referral OR “nutrition care” OR barrier OR facilitator* OR implement).
